# Evaluation of the effectiveness of alginate-based hydrogels in preventing peritoneal adhesions

**DOI:** 10.1093/rb/rbad017

**Published:** 2023-03-21

**Authors:** Zhu Meng, Han Wang, Yu Liu, Minyi Yang, Hang Zeng, Qianqian Han

**Affiliations:** National Institutes for Food and Drug Control, People’s Republic of China; National Institutes for Food and Drug Control, People’s Republic of China; School of Materials Science and Engineering, Beijing Institute of Technology, People’s Republic of China; National Institutes for Food and Drug Control, People’s Republic of China; YanTai University, People’s Republic of China; National Institutes for Food and Drug Control, People’s Republic of China; National Institutes for Food and Drug Control, People’s Republic of China; China Pharmaceutical University, People’s Republic of China; National Institutes for Food and Drug Control, People’s Republic of China

**Keywords:** peritoneal adhesions, sodium alginate hydrogel, mesothelial cells, TGF-β1

## Abstract

Infertility and intestinal blockage are just two examples of the postoperative consequences that can arise from peritoneal damage, which can also result in severe peritoneal fibrosis and peritoneal adhesions. Peritoneal adhesions are still not effectively treated, and both pharmaceutical therapy and biomaterial barriers have only had modest preventative effects. In this work, we looked into the effectiveness of in-place injectable sodium alginate hydrogel for peritoneal adhesion prevention. The findings demonstrated that sodium alginate hydrogel promoted human peritoneal mesothelial cell proliferation and migration, prevented peritoneal fibrosis by suppressing the production of transforming growth factor-β1, and, most importantly, promoted mesothelium self-repair. These findings imply that this brand-new sodium alginate hydrogel is a good candidate material for peritoneal adhesion prevention.

## Graphical abstract

**Figure:**
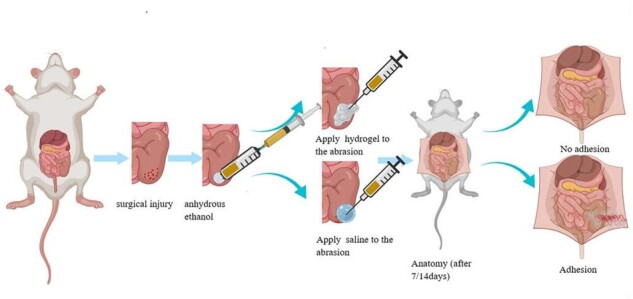

## Introduction

Adhesions are abnormal proliferations of fibrous tissue adhering to normal organs and are risks for various surgeries, such as abdominal thoracic pelvic adhesions [1], uterine adhesions [2], pericardial adhesions [3], epidural adhesions [4], joint ligament adhesions [5], etc. Up to 94% of individuals experience adhesion development following abdominal surgery, according to statistics [6]. An infection or bacterial infection brought on by extended contact to dust or air, ischemia brought on by excessive wound exposure or prolonged exposure to air, and foreign bodies of gauze debris left over during surgery can all produce inflammation that leads to peritoneal adhesions.

Adhesion-related diseases are the surgical problems linked to ‘adhesions’ [7]. There are many psychosocial issues that can seriously impair a person’s quality of life, including chronic abdominal or pelvic pain, digestive issues, extreme constipation or diarrhea, recurrent small bowel obstruction, female infertility, challenges with secondary surgery and a variety of other issues. According to a study by the International Adhesion Society (IAS), more than 67 000 Americans were hospitalized in 2001 due to an adhesive small bowel blockage, at a cost of more than $5 billion annually [8]. An estimated £569 million in direct costs have been attributed to adhesion-related readmissions in the UK 10 years after lower abdominal surgery, according to a figure from 2002 [9]. This represents a large financial burden.

Adhesions that form after abdominal surgery can be prevented using tried-and-true techniques, which not only save a tremendous amount of time and money but also free patients from recurrent problems.

Secondary open surgery or laparoscopy is still the standard treatment for peritoneal adhesions today [10, 11], although invasive adhesion release is associated with a high risk of readhesions. Clinical barriers and pharmacological treatment are the mainstays of postoperative adhesion prevention. Unfortunately, it is challenging to prevent adhesions due to the quick metabolism of anti-inflammatory or anticoagulant medications in the peritoneal cavity, which can even cause dangerous bleeding and issues with wound healing.

According to research [12], using adhesion barriers reduces the degree of adhesions and has been linked directly to a lower risk of postoperative morbidity following repeat cesarean surgery. Liquid anti-adhesion materials, solid anti-adhesion films and hydrogels are the main biomaterial barriers. Liquid anti-adhesion substances, like 4% Icodextrin [13], can diluted inflammatory factors, reduce inflammation and mesenchymal infiltration at the wound surface, and improve the ratio of mesothelial cells to mesenchymal infiltration, but they cannot play a long-term anti-adhesion role when adhesions are formed because it is difficult to maintain fluidity at the wound surface for a long time. Currently, medical absorbable anti-adhesion films [14] are most widely used both domestically and internationally, such as Seprafilm [15–17], Interceed [18] etc. Solid anti-adhesive film [19] has a physical barrier effect that can isolate inflammatory cells and inflammatory factors, and the effect is stable and long-lasting. However, it is challenging to cover irregular or large areas of injury, and the surgical exposure area is too large, so it is suitable for open surgery but not for minimally invasive surgery. Additionally, some materials are easily stuck to surgical instruments or other wet items, some need to be fixed with sutures, and some cannot be fixed. Hydrogel has both the fluidity of liquid anti-adhesive material and the stability of solid anti-adhesive film [20–22], which is a hot research topic in the field of biomaterials at present. Hydrogel, a prominent study issue in the field of biomaterials at the moment, combines the fluidity of a liquid anti-adhesive substance with the stability of a solid anti-adhesive film [20–22].

**Figure 1.**
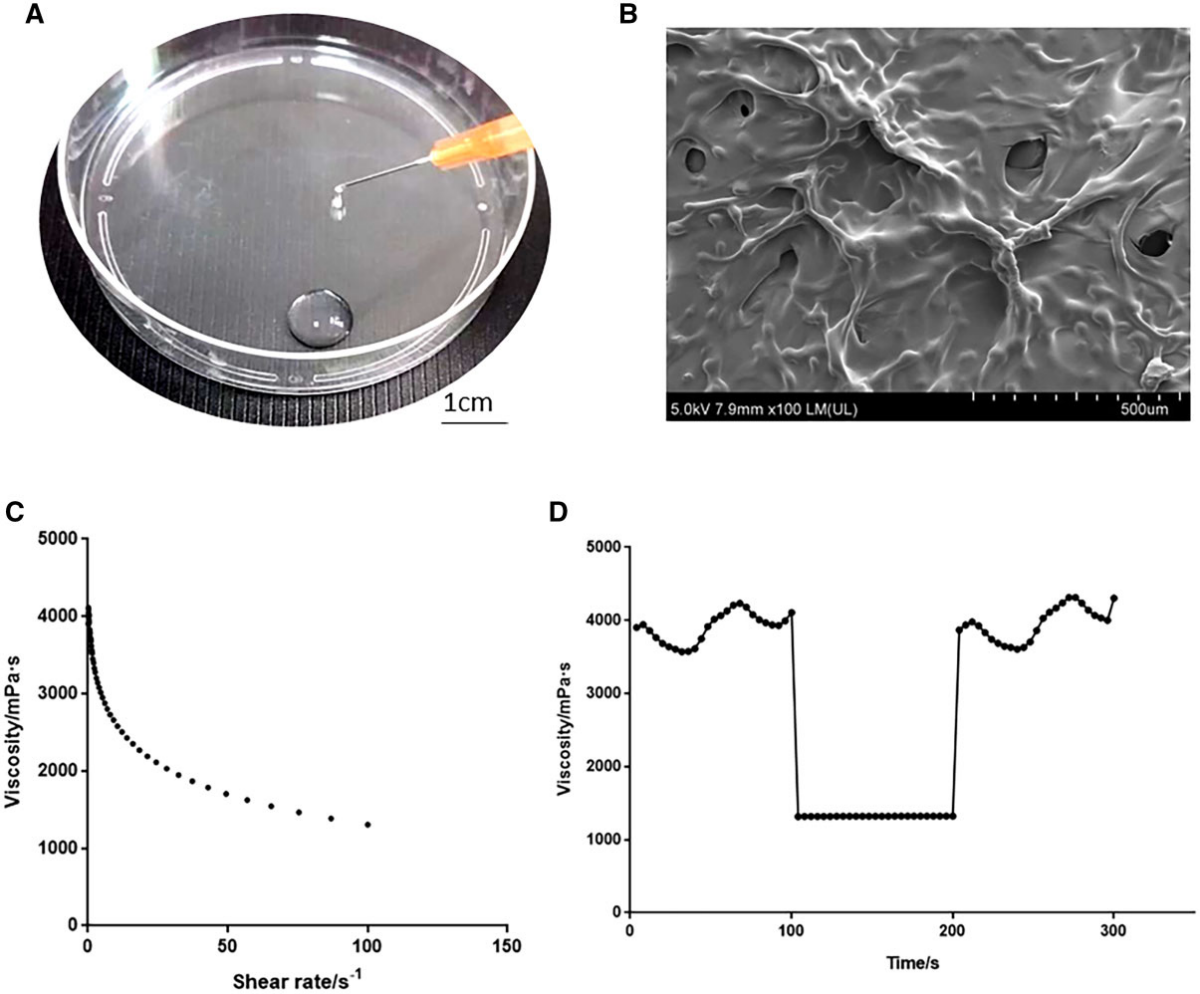
Characterization of SALG hydrogels. (**A**) SALG hydrogels passed through sterile syringe needle of 25G, scale bar: 1 cm. (**B**) SEM images of the SALG hydrogel. (**C**) Shear rate-sweep (0.1–1000 s^−1^). (**D**) Thixotropy-sweep.

In this study, sodium alginate was the primary component of an *in situ* injectable sodium alginate hydrogel, with some dextran added to improve the viscosity (Fig. 1A). This study aimed to assess the product’s efficiency in preventing peritoneal adhesions and serve as a guide for its therapeutic application.

## Materials and methods

### Materials

Human peritoneal mesothelial cells (HMrSV5) purchased from BNCC, Beijing, China. All cells were cultured in Dulbecco's modified eagle medium (DMEM) containing 10% fetal bovine serum (FBS) and 1% penicillin-streptomycin and incubated with 5% CO_2_ at 37°C. The Hangzhou Yingjian Biological Co., Ltd. developed sodium alginate (SALG) hydrogel, which is primarily made of sodium alginate and dextran. Medical Chitosan gel (Shijiazhuang Yishengtang Medical Products Co., Ltd, China), FBS (Gibco, USA), penicillin-streptomycin combined antibiotics (Gibco, USA), High Glucose DMEM (Gibco, USA), CCK-8 kit (Solarbio, China), PBS (Hyclone, USA), ROS reactive oxygen kit (Nanjing Jiancheng Institute of Biological Engineering, China), Propidium iodide dye kit (Keyueda, China) and Annexin V-FITC/PI apoptosis double-staining kit (BD, USA).

### Characterization of SALG hydrogels

Using a freeze-dryer (Martin Christ, Germany), the SALG hydrogels were dried for 24 h. After being sprayed with gold, the sample’s surface was examined under a scanning electron microscope (Hitachi, Japan). A rheometer (Anton-Paar, Austria) was used to measure the rheology of SALG hydrogels that were extruded from 25G sterile syringes at 25°C. Separate thixotropy and shear rate sweep tests were carried out. Each sample was put through a shear rate sweep test that ranged from 0.1 to 100 s^−1^. 0.1 s^−1^ shear rate for 100 s, 100 s^−1^ shear rate for 100 s and finally, 0.1 s^−1^ shear rate for 100 s were used in a ‘three-stage’ thixotropy sweep. From 0.1 to 100 s^−1^, the shear rate-sweep was carried out. The viscosity-shear rate curves ranged from 0.1 to 100 s^−1^.

### Animal experiment—rat cecum-abdominal wall adhesion model

**Figure 2.**
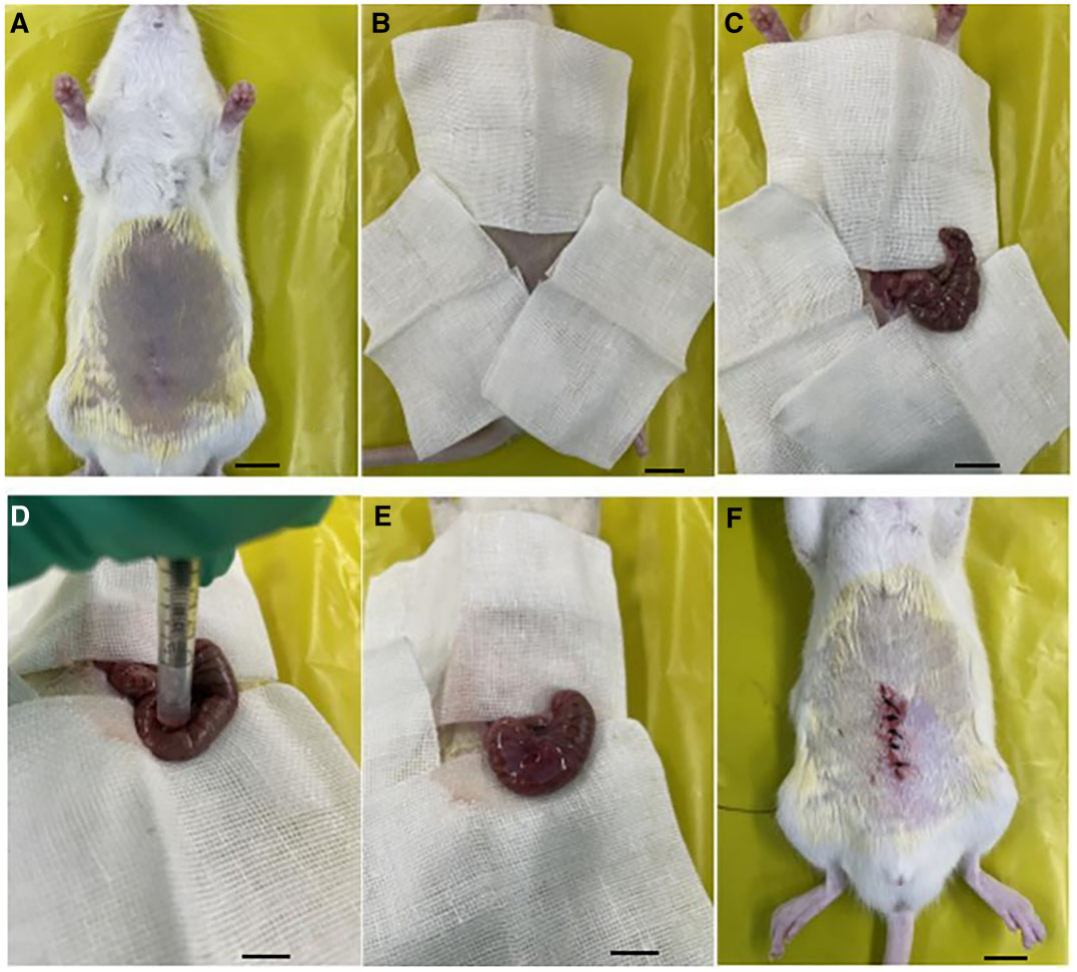
Diagram of the operation steps of the rat cecum-abdominal wall adhesion experiment, scale bar: 1 cm. (**A**) The lower abdomen of the rat is dehaired and cleaned with iodophor, while it is sedated and laying on its back. (**B**) Sterile towels are used to cover the surgery area. (**C**) The cecum is picked out and positioned in a uniform D-shape after the median line of the abdomen is sliced. (**D**) To cause chemical injury, the surface plasma membrane is scraped with sterile gauze 2–3 cm above the cecum’s root until punctate bleeding occurs. Next, 0.2 ml of anhydrous ethanol is injected into the cecum abrasion and left there for 30 s. (**E**) Apply either saline solution or a sample to the abrasion. (**F**) Close the abdomen and use 5–0 and 3–0 sutures, respectively, to stitch the skin and inner abdominal wall.

Forty-eight Sprague-Dawley (SD) rats of both sexes were obtained from the Animal Resource Center of the National Institute for Food and Drug Control, with experimental animal use permission No. SCXK (Beijing) 2022-0002. The rats were kept in individually ventilated cages at a humidity level of 40–70% and a temperature range of 20–25°C. The 48 rats were randomized into four groups: blank control group (Control Group), sham-operated group (Sham Group), chitosan gel group (Chitosan Group) and SALG hydrogel group (SALG Group). Fasting for 24 h before surgery, lower abdomen debridement, iodophor disinfection and sterile towel lying are all required. Anesthesia is administered via intraperitoneal injection of Zoletil (0.01 ml/kg). The rats were cut along the middle of the abdomen, the cecum was picked out, and the surface plasma membrane 2–3 cm above the root of the cecum was scraped with surgical sterile gauze until punctate bleeding, the area of gauze scraping was 1 × 2 cm^2^, the number of scraping was about 30–50 times to count the number of bleeding points and the number of bleeding points in each rat was kept as consistent as possible [23, 24]. A 1-ml syringe was used as a container and placed on the appendix abrasion. It was given a 0.2-ml injection of anhydrous ethanol to keep there for 30 s [25]. To cause chemical harm, the syringe was taken out and the anhydrous ethanol was blotted up with sterile gauze. The Control Group received no treatment, the Sham Group administered 0.5 ml of saline using a needleless syringe to the abdominal wall and cecum injuries, the Chitosan Group received 0.2 ml of medical chitosan gel, and the SALG Group received 0.2 ml of SALG hydrogel (the amount of the test group can make the animal tissue trauma coated to form a film of 0.1–0.2 mm thickness is appropriate). The abdominal wall and skin were stitched together with 5–0 sutures and 3–0 sutures, respectively, after the abdomen had been exposed to the air for 4 min. The whole process was operated as aseptically as possible. Figure 2 illustrates the experimental process.

Adhesion score following surgery. A group of animals were euthanized at 7 and 14 days after surgery, respectively. A ‘U’-shaped incision was used to incise the abdominal skin. After opening the abdomen, the adhesions of the abdominal wall and cecum were seen and graded using the Diamond [26] scale (Table 1).

**Table 1.** Adhesion scoring by Diamond

Characteristics	Adhesion scores
Degrees	0
None	1
≤25%	2
≤50%	3
>75%	4
Types	
None	0
Filmy, transparent, avascular	1
Semitranslucent or opaque, avascular	2
Opaque, obvious small vessels	3
Opaque, obvious larger vessels	4
Tenacity	
None	0
Adhesion easy to be separated	1
Adhesion to be separated by traction	2
Adhesion requiring sharp dissection for separation	3

### Pathology and immunohistochemical analysis

On Days 7 and 14 of the experiment, a group of rats was put to sleep, and the area of cecum adhesions in the abdominal wall of the rats in the Sham Group, SALG Group and Chitosan Group were excised. These tissues were embedded in paraffin and treated with 4% paraformaldehyde. The same area was then stained with Hematoxylin-Eosin (H&E) and transforming growth factor-β1 (TGF-β1) immunohistochemistry. A DP74 inverted microscope (Olympus, Japan) was used to observe the tissue sections. The appearance of brownish-yellow or brownish-brown granules in the cytoplasm was considered a sign of TGF-β1 positive.

### Blood biochemistry analysis

After 7 and 14 days of the experiment, 1 ml of whole blood was drawn from the abdominal aorta of rats in the SALG Group, Sham Group and Control Group, respectively. For blood biochemical examination using an automatic biochemical analyzer (Hitachi, Japan), 400 μl of the upper layer of the yellowish serum from each blood sample was removed during centrifugation at 7000 rpm.

### Cell proliferation, migration and adhesion detection

*Sample preparation*: SALG hydrogels were dissolved in 10% serum/serum-free medium containing and diluted multiplicatively to 50%, 25%, 12.5%, 6.25%, 3.125%, 1.5625%, and 0.78125% dilutions.

In 96-well plates, HMrSV5 was inoculated at a concentration of 1 × 10^3^/100 μl, and the plates were coated with high-glucose DMEM that contained serum. Twenty-four hours later, the plates were changed and co-cultured with SALG hydrogel containing serum-free medium dilution, and the serum-free group as control. The media was removed after 24 and 48 h, respectively, and a CCK-8 solution containing serum-free medium was added. The optical density (OD) values of Days 1, 3 and 7 were then evaluated using a SpectraMax M5 multifunctional enzyme marker (Molecular Devices, USA) to determine the viability of the cells.

Two horizontal lines were drawn down the middle of each well of a 24-well plate, 2 × 10^5^ HMrSV5 cells were placed in each well at a density of 700 μl, and the solution was changed after 24 h. With the serum group serving as a positive control, vertical lines were formed with a pipette tip, cleaned three times with PBS, and co-cultured with various multiples of SALG (25%, 12.5%, 6.25% and 3.125%) diluted in serum-free media. The serum-free group served as the study’s negative control. Pictures were shot at various times: 0, 12, 24, 36 and 48 h. CellSens Standard software was used to calculate the scratch area.

Six-well plates were coated with a thin layer of SALG hydrogel and chitosan gel, which was then sterilized for an hour using UV light. After plating 2 × 10^5^ HMrSV5 cells into each well of the 6-well plates, the media was removed to allow for double labeling with FITC-Phalloidin and DAPI after 24 h. The group receiving chitosan gel served as the control. Perform the test at least three more times.

The detection of reactive oxygen species (ROS) was done using a ROS detection kit. The sample fractions were diluted concentrations of 12.5% serum-containing and serum-free SALG hydrogel, while the control groups were serum-free medium and serum-containing medium. Before the test, cells were added to each sample group and cultured for an additional 24 h in 6-well plates at a density of 2 × 10^5^ cells/ml.

With the culture medium removed from the well plate, the 2,7-Dichlorofuorescin Diacetate (DCFH-DA) probe is directly diluted at a ratio of 1:1000, added to the serum-free medium, and then 1 ml of the diluted probe is added to each well. Set up a negative by adding only serum-free media and leaving out the probe. Thirty minutes of cell incubation at 37°C. To gather all the cells into a 1.5 ml centrifuge tube, aspirate out the medium, add 1 ml PBS and blow down several times. To completely remove the DCFH-DA probe that has not penetrated the cells, centrifuge with PBS at 1000 rpm for 5 min and wash twice. Finally, PBS was used to resuspend the cells in each tube, and the cells in each tube were counted to ensure that the cell concentration was constant throughout each tube.

The optimal wavelengths for excitation and emission were 500 and 525 nm, respectively. The outcomes were quantified using a multifunctional enzyme marker, the SpectraMax M5 and expressed as fluorometric values. Three times the experiment was conducted.

### Cell cycle and apoptosis assay

Six-well plates were prepared with 2 × 10^5^ HMrSV5 cells/ml, 2 ml of medium per well, and after 24 h the original medium was poured off and samples were added for co-culture in four groups, serum-containing and serum-free control group, 12.5% serum-containing and serum-free SALG hydrogel group. Cells were collected after 24 h. The assay was performed using the Propidium Iodide (PI) dye kit. The cells were treated according to the kit instructions and detected on flow cytometry (BD, USA). By labeling DNA with nucleic acid dye PI and analyzing it by flow cytometry, we can obtain the distribution status of cells at each period and calculate G0/G1%, S% and G2/M% to understand their proliferation capacity.

HMrSV5 was used to spread six-well plates at 2 × 10^5^/ml, and the procedure was performed as above. BD 556547 Annexin V-FITC/PI apoptosis double-staining kit was used. Phosphatidylserine (PS) is turned outward from the inner side of the lipid membrane at the beginning of apoptosis. A phospholipid-binding protein called Annexin V attaches to the PS that has been turned ectopically and acts as a sensitive indicator for the early identification of apoptosis. While late apoptotic cells and dead cells are marked red by propidium iodide (PI) due to enhanced cell membrane permeability, PI does not permeate the intact cell membrane. The cells were treated by the kit’s instructions. The sample was examined using a flow cytometer, and Flowjo software was used to quantify the percentage of viable, early apoptotic, late apoptotic and necrotic cells [27]. More than three times were done with the experiments.

### Statistical analysis

All data are expressed as $\bar{\text{x}}\;\;\pm \;\;\text{SD}$. One-way analysis of variance (ANOVA) was used to analyze the significance between three or more groups by GraphPad Prism. *P* < *0.05* was considered statistically significant.

## Results

### Injectability and stability of SALG hydrogels

As shown in Fig. 1A, the obtained hydrogel can easily pass through the 25G needle, and the extruded parts can be fused quickly and seamlessly. Meanwhile, the microstructure of the freeze-dried hydrogel was observed by scanning electron microscopy (Fig. 1B), and the uneven skeletons on the surface were interconnected in a 3D mesh structure with some toughness.

Using dynamic rheology, the shear thinning (Fig. 1C) and thixotropy (Fig. 1D) of this SALG hydrogel were analyzed. As the shear rate increased from 0.1 to 100 s^−1^, the viscosity gradually decreased, indicating that the hydrogel has the characteristic of shear thinning, which is beneficial to the shear extrusion of the gel. In the thixotropy experiment, the viscosity value of the first shear rate of 0.1 s^−1^ was used as the reference, and the viscosity return ratio was basically 100% when returning from 100 to 0.1 s^−1^ and this transition could be switched rapidly, indicating that the hydrogel has structural stability and rapid self-healing property.

### Gross evaluation of SALG hydrogel for inhibition of adhesions in rats

All 48 rats were alive after surgery. Regardless of the extent of the injured region [28], the peritoneum heals completely in 5–7 days when the mesothelium is disrupted and the peritoneum is mesothelialized again by mesothelial cells through a ‘bimodal mechanism’ [29]. Adhesion formation, therefore, depends on the first 5–7 days following surgery. As a result, we decided to look for adhesions on Day 7 and further confirm the results and the occurrence of adhesion advancement on Day 14.

**Figure 3.**
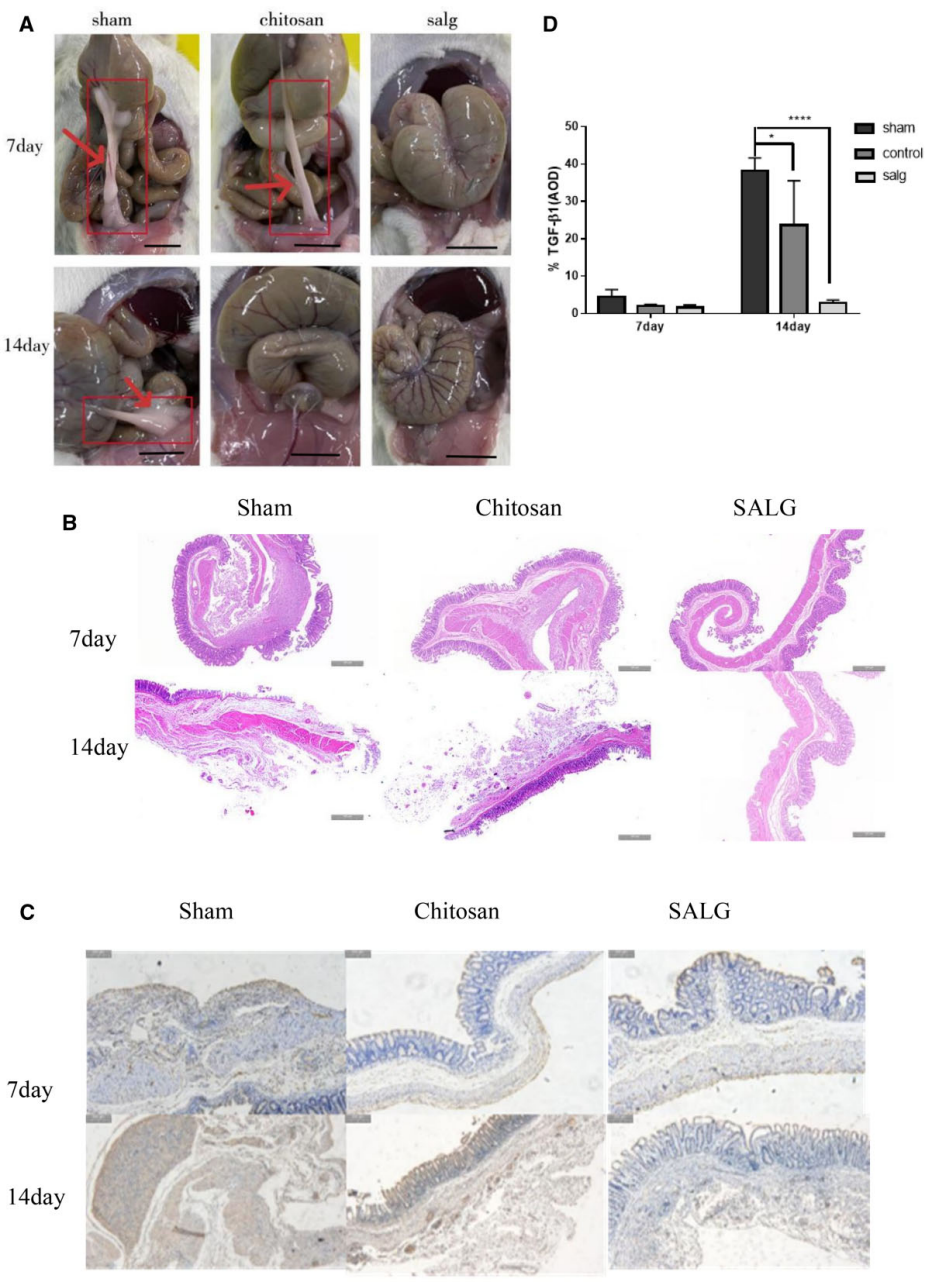
SALG hydrogel inhibits peritoneal adhesions in SD rats. (**A**) Tissue adhesions in different groups on postoperative days 7 and 14, scale bar: 1 cm (arrows are adhesion bands), *n* = 6. (**B**) H&E staining was performed on different groups of adherent tissues on postoperative days 7 and 14, scale bar: 500 μm, *n* = 6. (**C**) Immunohistochemistry for TGF-β1 was performed on the adhesions of different groups on the 7th and 14th day after operation, scale bar: 500 μm, *n* = 6. (**D**) The relative expression level of TGF-β1 protein was detected by immunohistochemical staining. *P < 0.05; ****P < 0.0001.

*Seven-day group*: six rats in the Sham Group had adhesions; three rats in the Chitosan Group had minor adhesions and three rats had no adhesions; three rats in the SALG Group had no adhesions and three rats had minor adhesions; *14-day group*: six rats in the Sham Group had adhesions; three rats in the Chitosan Group had no adhesions and three rats had adhesions, one of which was more serious and had extensive adhesions to the organs; four rats in the SALG Group had no adhesions and two rats had minor adhesions. Some of the test results are shown in Fig. 3A. The overall scoring results are shown in Supplementary Table S2.

According to the one-way ANOVA results (Table 2), there were significant differences in the incidence and severity of adhesions in the SALG Group and the Chitosan Group compared to the Sham Group after 7 days, and there was also a substantial anti-adhesion effect (*P* < 0.05). There was no significant difference between the SALG Group and the Chitosan Group, and the anti-adhesion effect of both groups was similar at 7 days. Analysis of the 14-day results showed a statistically significant difference in the incidence of adhesion inhibition and reduction of adhesion severity in the SALG Group compared with the Sham Group (*P* < 0.01). There was no significant difference between the SALG Group and the Chitosan Group. In summary, the SALG hydrogel has an anti-adhesive effect comparable to that of medical chitosan gel.

**Table 2.** Adhesion scores of each group according to Diamond scoring method ($\bar{\text{x}}\;\pm \;\;\text{SD}$)

Group Score	7 days	14 days
Sham Group	7.33 ± 0.88	6.67 ± 1.09
Chitosan Group	3.00 ± 1.34*	3.83 ± 1.76
SALG Group	2.83 ± 1.27*	1.16 ± 0.83**
Control Group	0****	0***

All data are presented as mean ± SD, *P < 0.05; **P < 0.01; ***P < 0.001; ****P < 0.0001

### SALG hydrogel inhibits peritoneal fibrosis and leaves the mesothelium intact

H&E staining [30] as seen in Fig. 3B, the 7-day Sham Group had a damaged plasma membrane surface, interrupted mesothelial cells, a large infiltration of inflammatory cells (lymphocytes, neutrophils, macrophages, etc.), a large number of fibroblasts proliferating outside the plasma membrane and connective tissue formation. The plasma membrane surface of the Chitosan Group was shattered, showing clear signs of inflammatory cell infiltration, small vessel formation and connective tissue proliferation. In the SALG Group, the plasma membrane layer was intact, the mesothelial cell layer was continuous, and there was no obvious inflammatory reaction. Fourteen-day group of Sham Group had a broken plasma membrane layer, no continuous mesothelial cell layer, reduced inflammatory cell infiltration, fibroblast proliferation and a large number of irregularly arranged collagen fibers formation. The Chitosan Group had a small amount of inflammatory cell infiltration, reduced fibroblast proliferation, a large number of collagen fibers loosely arranged and many thin-walled small blood vessels. The SALG Group was basically free of inflammatory cell infiltration, and the mesothelial layer was continuous and intact. In conclusion, the H&E staining results showed that SALG hydrogel reduced fibroblast proliferation, inhibited inflammatory cell infiltration, and promoted collagen fiber deposition, preserving the integrity of the appendix and speeding up wound healing.

### SALG hydrogel inhibits the expression of TGF-β1

In Fig. 3C and D, immunohistochemistry is displayed. One week after surgery, the adhesion site was extensive in the Sham Group, and TGF-β1 was expressed in fibroblasts and vascular endothelial cells in the plasma membrane layer of adhesions, and the adhesion area expanded and the positive distribution was diffusely enhanced after two weeks. The Chitosan Group expressed TGF-β1 positively mainly in the cytoplasm of fibroblasts in the plasma membrane layer after 7 days, and after 14 days the small vessels in the plasma membrane layer of the Chitosan Group proliferated, and TGF-β1 was positively expressed in the cytoplasm and nucleus of vascular endothelial cells as well as in the surrounding fibroblasts. The positive expression of TGF-β1 in the SALG Group was weak at 7 days, with brownish-yellow granules mainly in the mesothelial cell layer of the plasma membrane layer, and the positive expression of TGF-β1 was even weaker at 14 days. This indicates that SALG hydrogel can effectively reduce the expression of TGF-β1 in cecum tissue. TGF-β1 is one of the initiators of the inflammatory response and has a chemotactic effect on fibroblasts [31, 32]. According to the immunohistochemistry findings, SALG hydrogel may lessen fibroblasts’ proliferative chemotaxis, which had the impact of preventing adhesion deterioration.

### SALG hydrogel promotes proliferation, migration and adhesion of HMrSV5 cells

**Figure 4.**
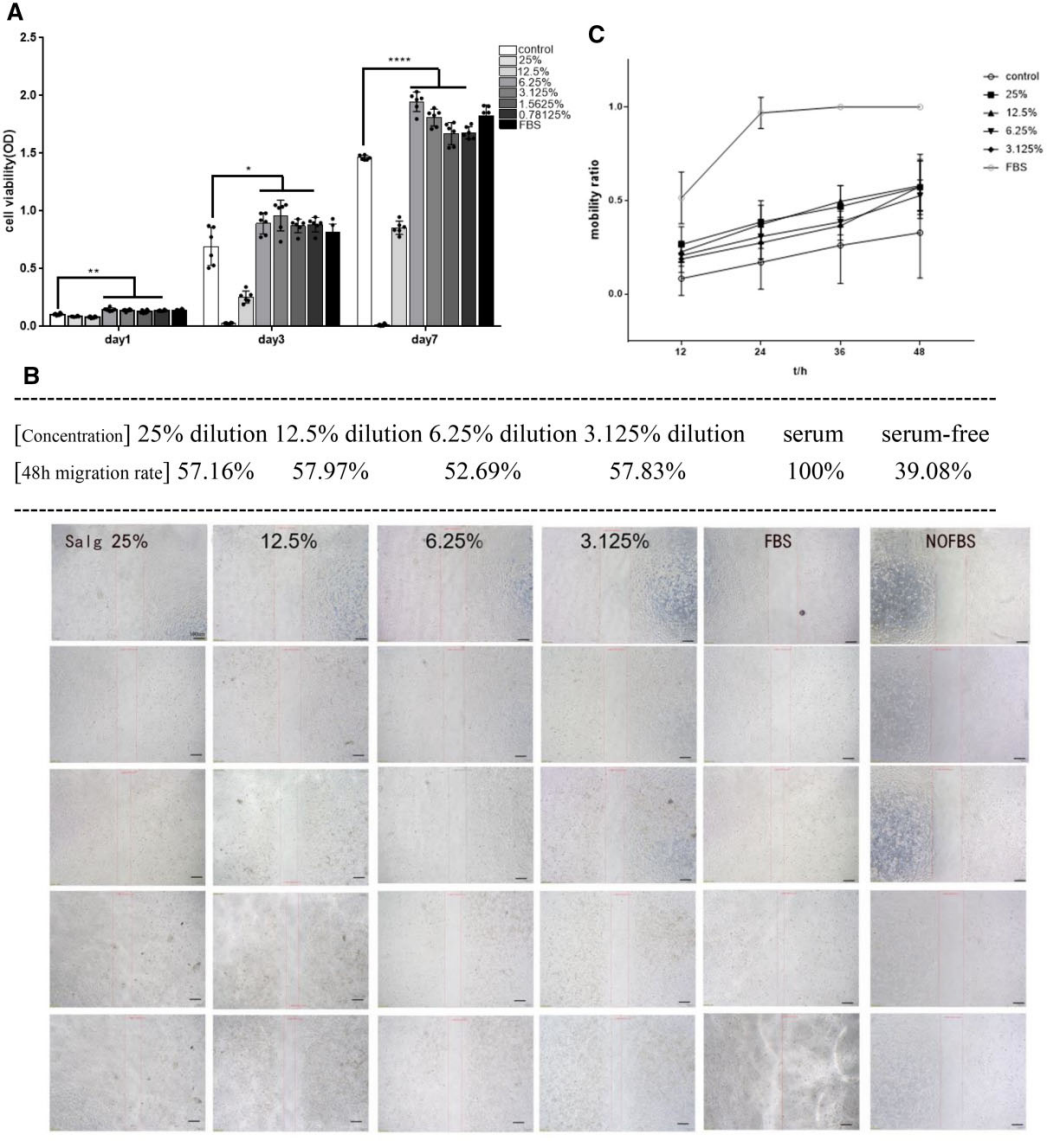
SALG hydrogel promotes the proliferation and migration of HMrSV5 cells. (**A**) Proliferation of HMrSV5 cells under co-culture with SALG hydrogel dilution (only one-way ANOVA results are indicated for sample groups not greater than 6.25% compared to the control group). (**B**) Schematic diagram of the scratches of HMrSV5 cells, scale bar: 50 μm, *n* = 4. (**C**) Cell migration rate of HMrSV5 cells in SALG hydrogel diluent. All data are presented as mean ± SD, *P < 0.05; **P < 0.01; ****P < 0.0001.

The processes of cell proliferation and apoptosis play a crucial role in the development of postoperative adhesions. The relevant literature shows that [33] peritoneal cells comprise 61.5% of mesothelial cells and 16.8% of fibroblasts. Therefore, we concentrated on researching the effectiveness of the samples on human peritoneal mesothelial cells. The cell proliferation viability of SALG hydrogel co-cultured with mesothelial cells (HMrSV5) is shown in Fig. 4A. The OD values of SALG hydrogels at dilution concentrations not greater than 6.25% were higher than those of the Control Group on Days 1, 3 and 5, and the differences were significant (*P* < 0.05), indicating the ability of SALG hydrogels at dilution concentrations ≤6.25% to promote the proliferation of HMrSV5 cells compared to the Control Group, as in the case of the serum group. This indicates that the hydrogel can considerably encourage mesothelial cell growth.

The cell migration results are shown in Fig. 4B and C. The cell migration rates of this SALG hydrogel were higher than those of the serum-free control group at the dilution concentrations of 3.125–25% and the cell migration rates of each group were very similar, indicating that this hydrogel has the effect of promoting cell migration.

**Figure 5.**
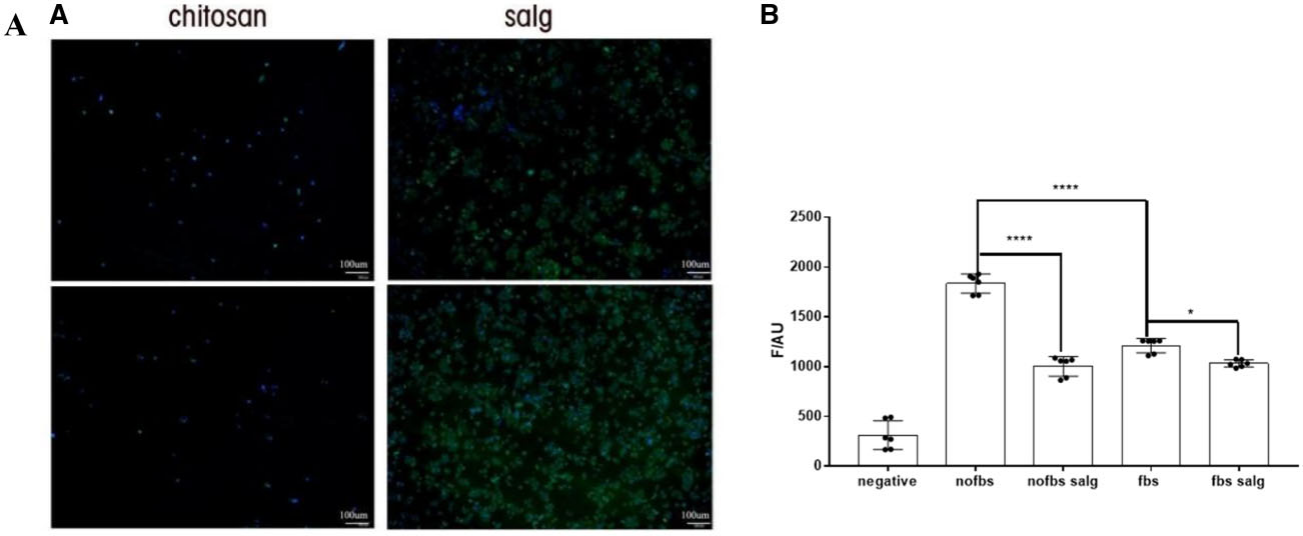
*In vitro* evaluation of the effectiveness of SALG hydrogel. (**A**) Adhesion of DAPI and FITC-Phalloidin double-stained HMrSV5 cells on chitosan gel and SALG hydrogel, scale bar: 50 μm. (**B**) Fluorescence values of reactive oxygen species under HMrSV5 co-culture with SALG hydrogel, *n* = 3 (all data are presented as mean ± SD, *P < 0.05; ****P < 0.0001).

The staining findings are compared in Fig. 5A, where it can be seen that mesothelial cells can develop on the SALG hydrogel but barely at all on the chitosan gel. The experiment demonstrates that the SALG hydrogel is superior to the chitosan gel for promoting the adhesion and development of human peritoneal mesothelial cells.

### SALG hydrogel inhibits the generation of reactive oxygen species

During the first 5 min of hypoxia in the body, free radicals are generated through increased production of ROS. Oxidative stress is hypothesized to be a susceptibility factor for the adherence phenotype because it results from an imbalance between the production of free radicals and antioxidant enzyme defense mechanisms [34]. In Fig. 5B, the outcomes of the ROS studies are displayed. Both the serum-free and serum-containing controls had significantly lower levels of reactive oxygen species after the addition of SALG hydrogel (*P* < 0.05). We come to the conclusion that SALG hydrogel can reduce oxidative stress in the body by preventing ROS from being produced.

### SALG hydrogel increased the proportion of HMrSV5 cells in G2/M phase and S phase, with no effect on apoptosis

**Figure 6.**
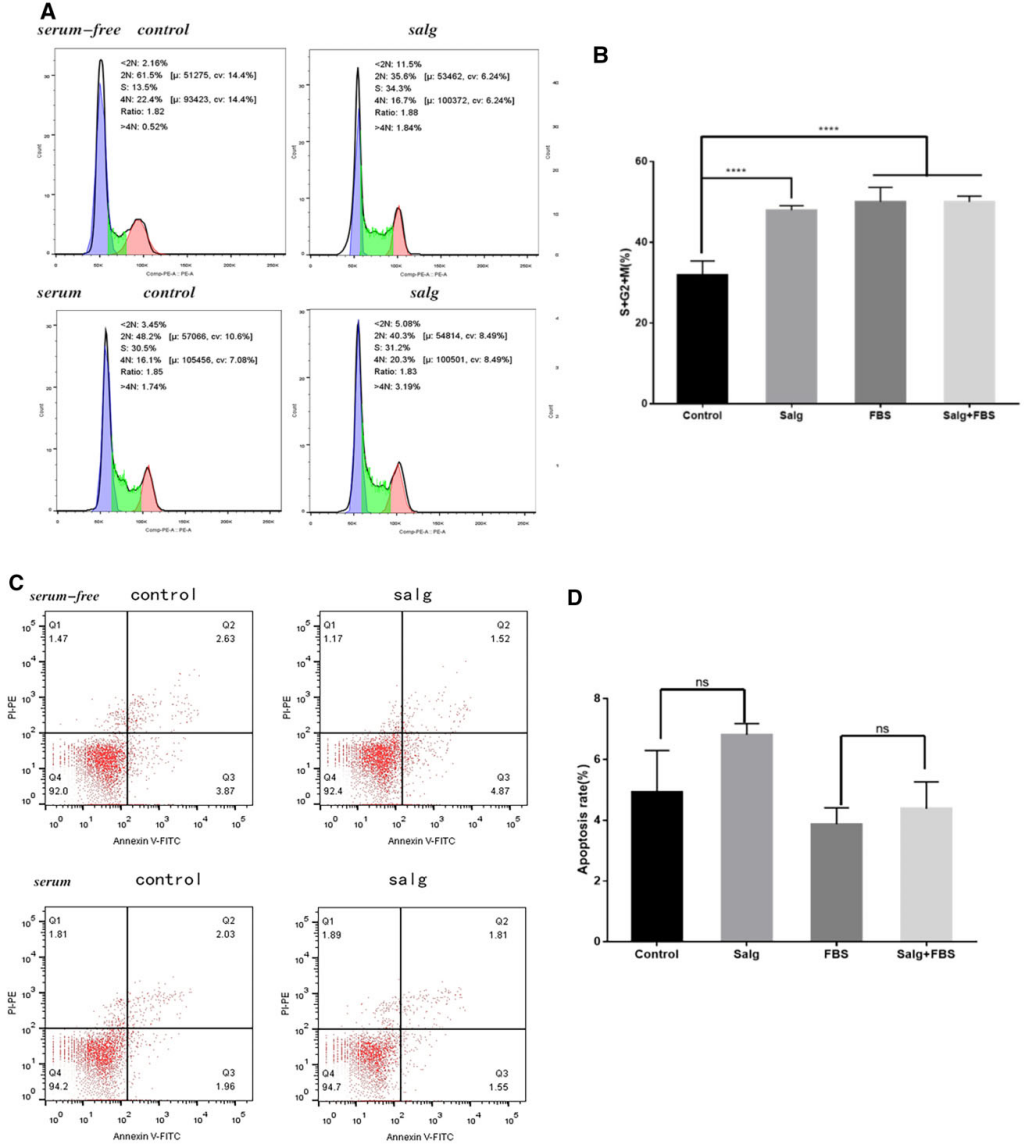
Cell cycle and apoptosis assays in SALG hydrogels. (**A**) Schematic diagram of cell cycle. (**B**) Effect of SALG hydrogel on the cell cycle of HMrSV5 cells for 24 h, *n* = 3. (**C**) Schematic diagram of apoptosis. (**D**) Effect of SALG hydrogel on apoptosis of HMrSV5 cells at 24 h, *n* = 3. All data are presented as mean ± SD, ****P < 0.0001; the ns means no significant difference.

To further explore the effect of SALG hydrogel-mediated proliferation of HMrSV5 cells, the changes in cell cycle and apoptosis after SALG hydrogel treatment were investigated by flow cytometry. The cell cycle results are shown in Fig. 6A and B. The S phase is the beginning of DNA synthesis, and the cells in the G2 phase continue to synthesize RNA and proteins until they enter the M phase. Therefore, G2/M%+S% reflects the cell proliferation capacity. The percentage of G2/M and S-phase cells significantly increased from 31.9 ± 3.46% to 47.95 ± 1.10% after serum-free SALG hydrogel treatment (*P* < 0.01). The percentages of the sum of S and G2/M phases in the serum-containing SALG hydrogel group were basically the same as those of the serum-containing control group, with no significant difference (*P* > 0.05). Statistical analysis also revealed no significant difference in the percentage of the sum of S and G2/M phases between the serum and serum-free SALG hydrogel groups (*P* > 0.05), indicating that SALG hydrogel alone exerted the same effect as serum in promoting DNA synthesis in HMrSV5 cells and promoting mesothelial cell proliferation [35].

Using a flow cytometer, the apoptosis rates are measured following Annexin V FITC/PI labeling (Fig. 6C). The percentages of Q2 + Q3, where Q2 stands for late apoptosis and Q3 for early apoptosis, are included in Fig. 6D. That is, total apoptosis. There was no significant difference in the apoptosis rate in the serum-free SALG hydrogel group compared to the serum-free control group (*P* > 0.05). There was no statistically significant difference between the serum-containing SALG hydrogel group and the serum group. It was shown that SALG hydrogel did not induce apoptosis in HMrSV5 cells. These findings support the safety of this hydrogel in clinical trials [36].

### *In vivo* blood biochemical analysis of SALG hydrogel

The following blood biochemical parameters were measured: alanine aminotransferase (ALT), aspartate aminotransferase (AST), triglyceride (TG), serum creatinine (Cre), serum albumin (ALB), total protein (TP), total cholesterol (CHO), urea (UREA), serum alkaline phosphatase (ALP) and trace elements such as sodium, potassium, calcium, phosphorus and chloride to assess the effects on liver function, kidney function, cardiac function, lipids, blood glucose, serum protein and electrolytes in rats.

**Figure 7.**
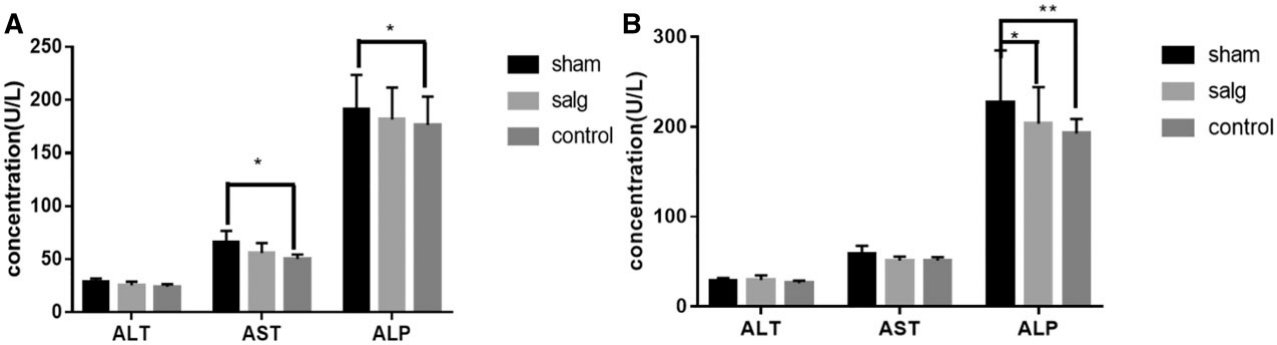
Statistical analysis of some biochemical indicators on the 7th (**A**) and 14th (**B**) postoperative days (all data are presented as mean ± SD, *n* = 6, *P < 0.05; **P < 0.01).

Some of the experimental results are shown in Fig. 7A and B. One-way ANOVA showed that compared with the Control Group, ALP and AST were both elevated to some extent in the Sham Group at 7 days, and there was a statistical difference (*P* < 0.05) between the Control Group and the Sham Group for AST and ALP, while all other indexes were not statistically different (Supplementary Table S1). At 14 days, compared with the Control Group, ALP was elevated to some extent in the Sham Group, and there was also a statistical difference in ALP between the Control Group and the Sham Group (*P* < 0.01). There was no statistically significant difference in ALP between the SALG Group and Control Group; however, there was one between the SALG Group and the Sham Group (*P* < 0.05).

Among them, ALT and AST are the most widely used biochemical indicators reflecting hepatocyte damage in clinical practice [37]. ALT is mainly distributed in the cytoplasm of hepatocytes and is a sensitive indicator of hepatocyte damage; AST is mainly distributed in the mitochondria of hepatocytes, and an increase in AST concentration may indicate that the hepatocytes are more heavily damaged, or that the mitochondria of hepatocytes are mainly invaded. ALP in hepatocytes is mainly bound to hepatocyte membrane and not easily released. ALP is an enzyme biomarker of bile stasis, and elevated serum ALP signals possible hepatobiliary illness. When the flow of bile is weak or bile stasis occurs, the backflow of ALP into the blood increases and the blood ALP rises dramatically. A number of blood biochemical indices (including ALP, AST and ALT) were normalized in the rats after the administration of SALG hydrogel, with no discernible change from the non-surgical Control Group. We found that the SALG hydrogel had good biocompatibility because there was no evidence of systemic toxicity of the hydrogel.

## Discussion

The ideal anti-adhesion hydrogel material should meet several basic requirements [38, 39] including good biocompatibility and biodegradability, excellent physical barrier properties, appropriate tissue adhesion and no interference with wound healing. In addition to hyaluronic acid [40, 41], chitosan [42, 43] and other commonly used anti-adhesive biomaterials, alginate has also shown potential anti-adhesive properties. Chaturvedi *et al*. [44] placed alginate gel or sodium hyaluronate-carboxymethylcellulose (HA/CMC) film on the anastomosis of rats after ileal anastomosis, respectively, to assess the effect on early healing of ileal high-risk anastomoses and found that the adhesion score was reduced by nearly 40% in the alginate gel group compared to the HA/CMC film group. Sodium alginate has mucosal adhesion properties in the solid state and becomes a viscous and slippery gel-like solution in the dissolved state. It is the target of our research due to its unique mucosal adhesion that allows it to reside in the wound without the need for sutures and its low inflammatory properties into the body [45].

This *in situ* injectable SALG hydrogel allows for precise fitting of irregularly defective tissue, complete coverage of the affected wound, and minimally invasive treatment. The hydrogel has a sugar content of 32.55% and suitable thickening properties to produce good stability and film formation. It has been found that sodium alginate has regeneration-promoting [46] antioxidant, antiviral [47] and resistance to radioactive damage [48]. The concentration of sodium alginate in this hydrogel is 25 mg/ml, and its wet contact environment [49] isolates and moisturizes the wound while absorbing wound exudate and minimizing secondary injury, achieving antibacterial, hemostatic and wound healing goals and having a ‘1 + 1 > 2’ effect.

TGF-β1 is a recognized major inducer of fibrosis and adhesion formation [31]. TGF-β1 is a critical inflammatory factor in the fibrosis phase of adhesion creation because it has chemotactic effects on fibroblasts, and stimulates the proliferation and differentiation of inflammatory cells, as well as angiogenesis [32]. The hydrogel not only blocked inflammatory factors and inflammatory cells by functioning as a physical barrier, but it also inhibited fibroblast aggregation and peritoneal fibrosis by suppressing TGF-β1 expression in cecum tissue from rats with cecum-abdominal wall abrasion, which was shown by immunohistochemistry of TGF-β1. H&E staining further revealed that it promoted the repair of the peritoneal mesothelium and contributed to the healing of the damaged peritoneum.

Mesothelial cells play a prominent role in peritoneal damage [50, 51]. The peritoneum is a smooth, translucent, semi-permeable membrane in the peritoneal cavity, mainly covered by a single layer of flat, microvilli-rich mesothelial cells [52–54]. When the peritoneum is severely damaged, a local inflammatory response is triggered and inflammatory factors such as TGF-β1 are produced in large quantities, inducing a decrease in tissue-type fibrinogen activator (tPA) synthesis and an increase in fibrinogen activator inhibitor (PAI-1) release by peritoneal mesothelial cells [55], resulting in an elevated balance of the fibrinolytic system and a mesothelial–mesenchymal transition [56], which gradually forms a fibroblast-like phenotype and further synthesizes secreted extracellular matrix components (ECM), such as type I collagen, fibronectin or elastin [57, 58], which are involves in collagen deposition. When fibroblasts infiltrate these extracellular matrices, they create long-lasting fibrous adhesions.

Human peritoneal mesothelial cells (HMrSV5) were tested *in vitro* for cell proliferation, migration, adhesion, cell cycle, apoptosis and the presence of reactive oxygen species. It was discovered that this SALG hydrogel aided in these processes while having no effect on apoptosis and inhibiting the production of ROS. Additional blood biochemical testing revealed that the SALG hydrogel had great biocompatibility and did not affect the liver, kidneys or electrolytes in rats following laparotomy.

This study confirmed the *in situ* injectable SALG hydrogel’s effective anti-adhesive properties at both the cellular and animal levels. In conclusion, we showed that this SALG hydrogel supports the growth of mesothelial cells and promotes mesothelium self-repair by offering a proper environment for mesothelial cell growth.

## Conclusion

Currently, the physical protective effect of biological materials’ anti-adhesion barriers is mostly considered, with the impact of peritoneal healing receiving little attention. Instead of mesothelial cells, it primarily focuses on the evaluation of the materials’ effects on fibroblasts. The study showed that the SALG hydrogel has excellent injectability, stability and quick self-healing properties, good biocompatibility and mesothelial cell adhesion, and can support human peritoneal mesothelial cell proliferation and migration while reducing the production of reactive oxygen species. As a result, it may be said that the hydrogel prevents abdominal adhesions. According to the aforementioned findings, this alginate-based anti-adhesion hydrogel may be used in clinical settings as a medical device. Additionally, this study offers fresh design and research concepts for the creation of innovative biomaterials that do not adhere to the peritoneum.

## Supplementary Material

rbad017_Supplementary_Data
