# Supplementary material for: Evaluation of the effectiveness of alginate-based hydrogels in preventing peritoneal adhesions
Source: Regen Biomater. 2023 Mar 21;10:rbad017. doi: 10.1093/rb/rbad017 (PMC10110266; doi:10.1093/rb/rbad017)
Supplement: rbad017_Supplementary_Data [file rbad017_supplementary_data.docx]

| Projects | 7day | | | 14day | | |
| --- | --- | --- | --- | --- | --- | --- |
|  | Control | SALG | Sham | Control | SALG | Sham |
| ALT（U/L） | 28.00±3.35 | 25.50±3.10 | 24.00±2.16 | 28.67±2.62 | 29.67±4.68 | 26.20±2.19 |
| AST（U/L） | 65.80±9.77 | 55.83±8.59 | 50.33±3.40* | 58.00±7.79 | 51.00±4.28 | 51.00±3.01 |
| AST/ALT（%） | 2.35±0.25 | 2.19±0.22 | 2.11±0.24 | 2.05±0.41 | 1.75±0.26 | 1.98±0.27 |
| ALP（U/L） | 190.80±29.44 | 181.83±27.36 | 176.67±21.75  * | 226.67±47.79 | 203.50±37.38 | 192.50±11.50  ** |
| GLU（mmol/L） | 9.91±0.52 | 10.28±1.73 | 10.57±1.34 | 10.85±1.08 | 9.89±1.27 | 18.40±5.26 |
| UREA（mmol/L） | 6.06±0.96 | 6.06±0.52 | 9.40±0.93 | 6.45±0.92 | 6.82±0.96 | 7.78±0.13 |
| CHO（mmol/L） | 1.48±0.142 | 1.67±0.13 | 1.69±0.11 | 2.05±0.17 | 1.70±0.26 | 1.76±0.05 |
| TP（g/L） | 49.32±2.87 | 51.27±3.59 | 55.33±6.22 | 58.83±3.43 | 58.17±3.31 | 55.80±0.10 |
| ALB（g/L） | 21.16±0.95 | 21.32±0.18 | 22.98±1.86 | 24.03±1.71 | 24.78±1.46 | 22.59±0.50 |
| Ca（mmol/L） | 2.48±0.13 | 2.63±0.18 | 2.70±0.16 | 2.50±0.24 | 2.63±0.31 | 2.90±0.30 |
| P（mmol/L） | 2.51±0.077 | 2.65±0.41 | 2.39±0.27 | 2.32±0.10 | 2.46±0.25 | 2.95±0.20 |
| Cl（mmol/L） | 100.19±0.79 | 100.58±0.75 | 101.58±0.75 | 101.98±0.56 | 101.35±1.40 | 100.85±0.84 |
| CRE（mmol/L） | 22.20±1.60 | 23.50±3.45 | 25.67±1.70 | 26.67±2.49 | 25.00±2.16 | 33.50±0.51 |
| K（mmol/L） | 4.62±0.26 | 4.40±0.74 | 4.13±0.26 | 4.00±0.16 | 4.53±0.63 | 6.45±1.15 |
| TG（mmol/L） | 0.64±0.08 | 0.74±0.13 | 0.68±0.16 | 0.78±0.08 | 0.81±0.26 | 1.21±0.44 |
| Na（mmol/L） | 147.66±0.68 | 148.17±0.77 | 151.97±1.82 | 150.63±0.59 | 150.70±1.84 | 150.65±1.85 |

Notes: * indicates comparison with the control group, P<0.05, ** indicates comparison with the control group, P<0.01.

Supplementary Table S1

| Time  Group | Day7 | | | | | | Day 14 | | | | | |
| --- | --- | --- | --- | --- | --- | --- | --- | --- | --- | --- | --- | --- |
| Sham Group | 9 | 8 | 8 | 3 | 8 | 8 | 6 | 7 | 4 | 4 | 11 | 8 |
| Chitosan Group | 6 | 0 | 6 | 5 | 0 | 0 | 0 | 2 | 5 | 0 | 0 | 0 |
| SALG Group | 0 | 6 | 0 | 6 | 6 | 0 | 0 | 6 | 7 | 0 | 0 | 8 |
| Control Group | 0 | 0 | 0 | 0 | 0 | 0 | 0 | 0 | 0 | 0 | 0 | 0 |

Supplementary Table S2
